# Theoretical Hill-Type Muscle and Stability: Numerical Model and Application

**DOI:** 10.1155/2013/570878

**Published:** 2013-11-12

**Authors:** S. Schmitt, M. Günther, T. Rupp, A. Bayer, D. Häufle

**Affiliations:** ^1^Department of Sports and Exercise Science, University of Stuttgart, Allmandring 28, 70569 Stuttgart, Germany; ^2^Stuttgart Research Centre for Simulation Technology, University of Stuttgart, Pfaffenwaldring 5a, 70569 Stuttgart, Germany; ^3^Institute of Sports Science, Science of Motion, University of Jena, Seidelstraß 20, 07749 Jena, Germany

## Abstract

The construction of artificial muscles is one of the most challenging developments in today's biomedical science. The application of artificial muscles is focused both on the construction of orthotics and prosthetics for rehabilitation and prevention purposes and on building humanoid walking machines for robotics research. Research in biomechanics tries to explain the functioning and design of real biological muscles and therefore lays the fundament for the development of functional artificial muscles. Recently, the hyperbolic Hill-type force-velocity relation was derived from simple mechanical components. In this contribution, this theoretical yet biomechanical model is transferred to a numerical model and applied for presenting a proof-of-concept of a functional artificial muscle. Additionally, this validated theoretical model is used to determine force-velocity relations of different animal species that are based on the literature data from biological experiments. Moreover, it is shown that an antagonistic muscle actuator can help in stabilising a single inverted pendulum model in favour of a control approach using a linear torque generator.

## 1. Introduction

Research in muscle biomechanics, a vital and broad field for over 80 years now (A.V. Hill 1922: Nobel prize in physiology and medicine for his discovery relating to the production of heat in the muscle), explains the function and design of real biological muscles and therefore lays the fundament for the development of functional artificial muscles. Nevertheless, structure and functioning of biological muscles are not (yet) fully understood.

In biology, microscopic muscle models are able to predict muscle characteristics and functioning of biological muscles quite well [1–9]. Unfortunately and as a tradeoff, they require a large number of parameters. In a bionics approach it is an enormous challenge to realise all these properties of biological muscle in one artificial muscle at once [10].

Macroscopic muscle models are commonly based on phenomenology. Macroscopic muscle models are indeed of (limited) predictive character but do not incorporate any structural knowledge. Recently, the nonlinear (hyperbolic-like) Hill-type force-velocity relation was derived from simple mechanical components [11]. It was shown that a contractile element (CE) consisting of a mechanical energy source (active element AE), a parallel damper element (PDE), and a serial element (SE) exhibits operating points with nonlinear (hyperbola-like) force-velocity dependency. In this concept, the force-velocity relation is no longer a phenomenological outcome of a black box (i.e., the CE) but rather a physical outcome of the interaction of the three elements AE, PDE, and SE. Based on this concept, it is now possible to describe in detail which structural arrangement is necessary to get a biology-like force-velocity relation on a macroscopic level. Therefore, this concept can be interpreted as a basic engineering design for the CE of a Hill-type artificial actuator [12–15]. In this paper, the meaning of the structural arrangement of the simple mechanical components already published will be revisited. Furthermore, by taking one first example of technical embodiment, it will be shown how this concept can help to construct more biologically-motivated artificial muscles. Most importantly, it can be shown that the application of a Hill-type muscle model could improve biomechanical stability. An antagonistic pair of our muscle model theoretically shows a first demonstration of how an artificial muscle could help in the stabilisation of a technical machine. As a result, the control of an inverted pendulum can be improved by the use of a muscle-like drive in favour of a linear torque generator.

## 2. Material and Methods

### 2.1. Hill's Original Formulation of Muscle Dynamics

In his famous paper “The heat of shortening and the dynamic constants of muscle” [16], Hill firstly formulated the simple and convenient equation describing the muscle's contraction dynamics:

(1) $$\begin{array}{c} \left(P+a\right)\cdot v=b\cdot \left(P_{0}-P\right), \end{array}$$

where the symbol “*P*” denotes the current muscle force, “*v*” the respective contraction velocity of the muscle, “*P*_0_” the muscle's maximum isometric force, and “*a*” and “*b*” Hill's so-called dynamic constants of muscle, which we call the Hill parameters.

### 2.2. Derivation of the Hill Parameters

**Figure 1:**
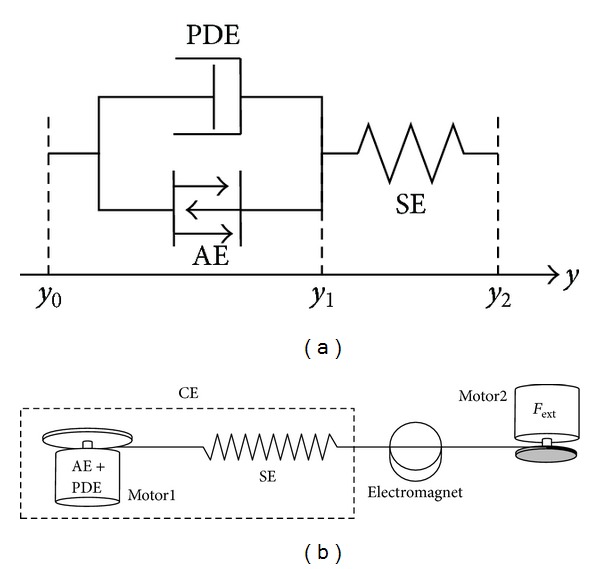
(a) Theoretical construct of the CE [11]. The CE consists of three elements: active element AE, parallel damping element PDE, and serial element SE. *y*_0_ = 0 is the origin of the CE, *y*_1_ the length of the AE/PDE, and *y*_2_ the length of the whole CE. By choosing *κ*_*v*_ = 0.0 in theory, we can turn the SE off in order to represent a contractile element without any compliance. (b) Hardware design. AE and PDE were realised with an electric motor, SE with a mechanical spring. A second electric motor was used to exert a defined external force *F*_ext_ on the CE. The electromagnet held the muscle at constant length until release at time *t* = *t*_QR_.

In a recent paper [11] it was demonstrated that the phenomenologically found hyperbolic force-velocity relation of a concentrically contracting assembly of activated muscle fibres [16] can be derived from the simple mechanical arrangement (Figure 1(a)) of an arbitrary force generating (active) element (AE) to which a damper (PDE) is connected in parallel and a serial element (SE) in series fulfilling the force equilibrium:

(2) $$\begin{array}{c} F_{\text{CE}}=F_{\text{SE}}=F_{\text{AE}}+F_{\text{PDE}}, \end{array}$$

where the symbol “*F*” denotes a force produced by the element denoted by a corresponding index and the kinematic relation for the lengths (symbols “*l*”) of the elements AE, PDE, and SE:

(3) $$\begin{array}{c} l_{\text{AE}}=l_{\text{PDE}}=l_{\text{CE}}-l_{\text{SE}} \end{array}$$

with *l*_CE_ representing the contractile element length. Note that a dot symbol “$\dot{l}$” denotes the first time derivative of a length *l*, that is, an element's contraction velocity. Please refer to the appendix or [13, 15] for a more detailed description.

### 2.3. Technical Embodiment

The hardware implementation (Figure 1(b)) was done analogously. Both AE and PDE were realised each with an electric motor (Maxon ECmax40) [14]. The motor torque (*T*_Motor_) was controlled by Maxon digital EC-motor control units (DEC 70/10). Both motors were mounted from opposite sides to the same disc with the radius *r*_disc_ = 0.05 m. The disc was used to coil up a steel rope and exert a force

(4) $$\begin{array}{c} F_{\text{AE}}+F_{\text{PDE}}=\frac{1}{r_{\text{disc}}}\cdot \left(T_{\text{MotorAE}}+T_{\text{MotorPDE}}\right) \end{array}$$

on the rope. The force characteristics of the PDE and AE ((A.1)) and ((A.4)) were implemented in MATLAB Simulink through Real-Time Workshop and Real-Time Windows Target. Thus, the motors could exert the specified force on the steel rope as required by the theoretical construct. For the SE, a spring (*k*_SE_ = 2401 Nm^−1^) was tied to a steel rope. Another motor could exert a defined external force on the CE construct. As sensor signals, the motor shaft positions *φ*_Motor_ were recorded by optical encoders (Scancon 2RMHF 5000 pulses/revolution), representing the internal degree of freedom *y*_1_ and the total CE length *y*_2_. A load cell (Transducer Techniques MLP 25 with amplifier TM0-1-24) was used to calibrate motor torques and exerted forces. All sensor data were recorded with MATLAB Simulink via a Sensoray 626 AD I/O at 1 kHz.

To investigate the force-velocity characteristics of the artificial CE, two types of experiments had to be performed. The first experiment was an isometric contraction (contraction with constant CE length: *y*_2_ − *y*_0_ = const.). For this purpose, the CE end was fixed to the electromagnet guaranteeing a constant CE length. Then the AE activation was set to *A*_AE_ = 1 (maximum activation) and the shortening of the AE (rotation of the motors) was recorded. The time from the beginning of the activation until the end of AE shortening *t*_isom_ and the maximum isometric force *F*_CE_(*t*_isom_) = *F*_CE,max⁡_ were evaluated.

Isotonic quick release experiments were performed to guarantee a defined *κ*_*v*_ = 0 (velocity ratio; please see appendix for a definition of *κ*_*v*_). Each isotonic quick release contraction experiment started like an isometric contraction, only that the CE was released at *t*_QR_ > *t*_isom_ (*t*_QR_ = 3 s) by releasing the electromagnet. CE contraction velocity and force were evaluated shortly after *t*_QR_ at *t*_eval_ = 3.5 s. The values *v*_CE_(*t*_eval_) and *F*_CE_(*t*_eval_) were extracted. The experiment was performed with different external forces, ten repetitions each. The curve *F*_CE_(*t*_eval_) versus *v*_CE_(*t*_eval_) for all external forces represents the force-velocity characteristics of the artificial CE (Figure 3(a), crosses).

### 2.4. Representing the Variety of Biological Muscles

In a further evaluation of our theoretical approach we scaled the model parameters to represent various biological muscles of different animals (Figure 3(b)). The model parameters *R*_PDE_ and (1 − *κ*_*v*_)*D*_PDE_ were calculated ((A.6)) from *A* and *B* values determined in experiments for different biological muscles (Table 1).

### 2.5. Control of the Inverted Pendulum

**Figure 2:**
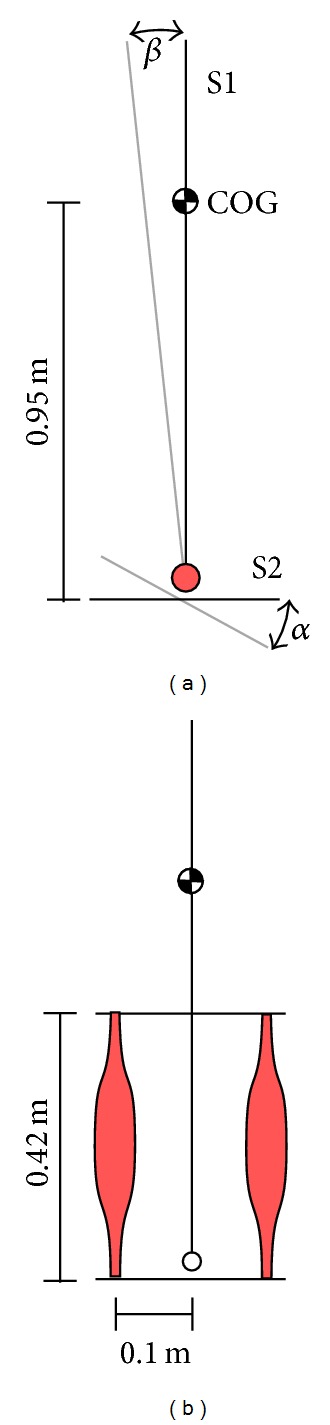
Model of the inverted pendulum. S1 represents the leg-trunk segment; S2 represents the foot. COG indicates the center of gravity location of S1. *α* is the angle of the foot (perturbation) and *β* the deviation from the upright position of S2. (a) The joint is actuated by a direct torque generator with linear characteristics. (b) The joint is actuated by two antagonistic muscles.

A model of an inverted pendulum was used to investigate what effect muscle-like actuator characteristics could have on the control of robotic stance. For quiet stance, the task was to keep an upright posture while deflecting the ground to which the pendulum was suspended with a hinge joint. The model consisted of two rigid segments connected with a hinge joint (Figure 2). S1 had a mass of *m* = 50 kg, a moment of inertia of *J* = 45.125 kgm^2^ (calculated around hinge joint axis), and the center of gravity was at *h*_COG_ = 0.95 m. The initial orientation of the leg/trunk segment S1 was vertical and horizontal for the foot segment S2. The pendulum could be perturbed by rotating S2 about the joint by the angle *α*. Three perturbations were considered: (a) a linear ramp increase of *α* = 1° · *t* ≤ 1° (for 0 ≤ *t* ≤ 1, where *t* is the time) and *α* = 1° (*t* > 1), (b) a sinusoidal oscillation *α* = 1° · sin(2*πt*), and (c) a sinusoidal oscillation *α* = 1° · sin(0.2*πt*).

The hinge joint could be actuated either by a direct torque generator or by an antagonistic pair of muscles (Figures 2(a), and 2(b)). The muscles were represented by two macroscopic muscle models of the same type. These muscle models incorporate the contraction dynamics, as well as a serial and a parallel elastic element representing the tendon and the passive elastic properties of soft muscle tissue. The muscle model was already described in detail in [11]. The parameters used for the muscle models are listed in Table 2. Both muscles were connected to a simple geometry as depicted in Figure 2.

Muscles and direct torque generator were controlled based on a feedback signal measuring the deviation of segment S1 from the vertical orientation. A physiological delay of Δ*t* = 0.1 s was considered. Three different controllers were applied: (1) no feedback is provided, (2) a simple proportional feedback (P controller), and (3) a PID controller. MATLAB Simulink embedded ODE5 (Dormand-Prince) solver with 1 ms step size was used to solve the differential equations.

## 3. Results

**Figure 3:**
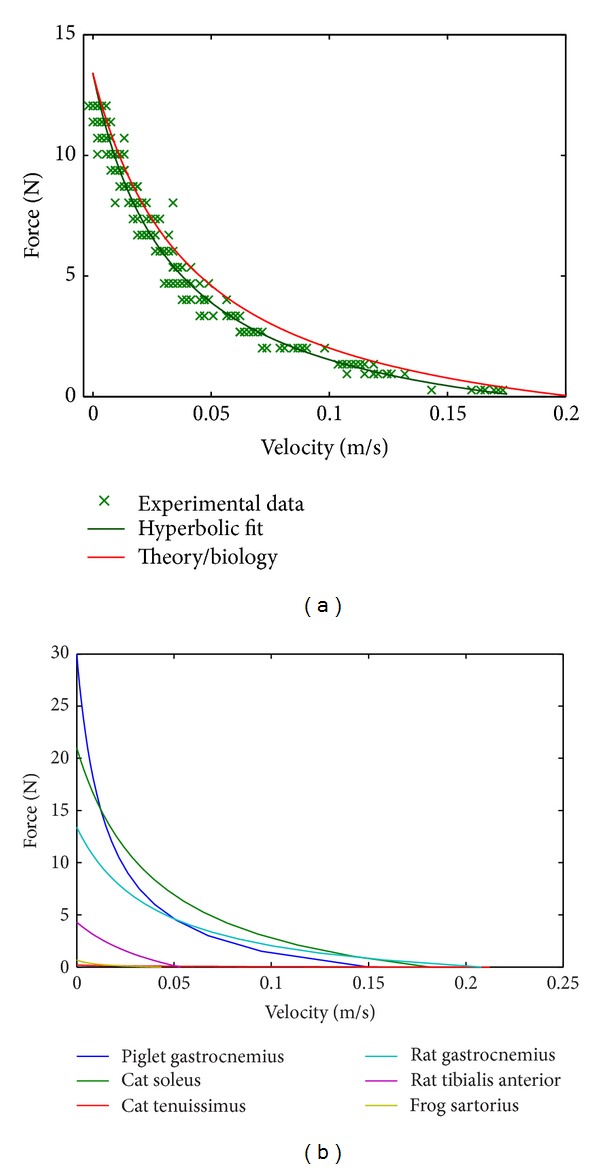
(a) Ten *F*(*t*) and *v*(*t*) plots for quick-release contraction experiments using 19 different external forces were recorded. Based on those *F*(*t*) and *v*(*t*) plots the force-velocity curve (crosses) was calculated. In direct comparison with the biological experiments (rat gastrocnemius muscle [20]) and the predictions from theory, the artificial muscle data shows a good match for both, with *κ*_*v*_ = 0. A hyperbola fit of the artificial muscle data results in *R* ^2^ = .97 [13, 15]. (b) The strength of the presented approach is shown by a comparison of *F*(*v*) curves calculated for different biological muscle parameters. The respective *F*-*v* curve can be plotted by just taking *A* and *B* values from experiments and calculating the parameters *R*_PDE_ and (1 − *κ*_*v*_)*D*_PDE_. The latter two parameters are necessary to build a technical muscle of that respective type.

 The relation between muscle output force and its contraction velocity is the common criterion for the comparison of macroscopic muscle models. Therefore, we calculated the *F*-*v* curve (Figure 3(a)) first. The *F*-*v* curve of the presented functional artificial muscle shows a very good match with both the prediction from theory and biological experiments.

By comparing our artificial muscle prototype's force-velocity relation as shown above, we consider our approach as quite successful. The functional artificial muscle prototype exhibits contraction dynamics similar to Hill's model characteristics (Figure 3(a) [13, 15]).

**Figure 4:**
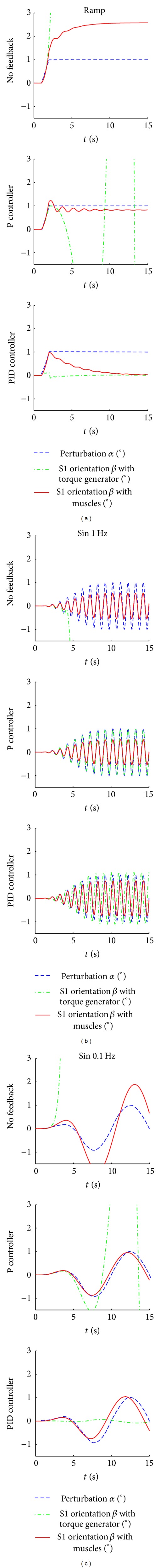
Model reactions to perturbations in foot orientation *α*. Control target is the upright posture (*β* = 0°). Left column shows the reaction to a ramp perturbation, middle column to a 1 Hz, and right column to a 0.1 Hz sinusoidal perturbation. Top row is without feedback, middle row with a simple P controller (direct torque controller gain: P 500; muscle controller gain: P 1), and bottom row with a PID controller (direct torque controller gains: P 500, I 50, D 500; muscle controller gains: P 1, I 0.3, D 0.3). The feedback signal is delayed by Δ*t* = 0.1 s.

In a model of the inverted pendulum, muscle-like nonlinear actuator characteristics were compared against a direct torque generator (linear characteristics). The muscle-driven model did not topple, not even without feedback (first row, Figure 4). Also, when using the simple P controller (middle row) the muscle-driven model performed better during all perturbations and was able to cope better with the feedback delay of Δ*t* = 0.1 s. Using the PID controller solutions were only found, where the direct torque controller performed better (bottom row, Figure 4). Here, a solution with high gain for the integral part of the PID controller is presented. Therefore, slow perturbations could be compensated very effectively.

## 4. Discussion

### 4.1. Element Representation

A brushless dc electric drive was used for the active element (AE) which was formulated in theory [11]. The tradeoffs of these actuators are the torque-to-weight ratio and the necessity of a power supply, either over cable or by battery. Madden [22] gives an overview of the current state of the art of technical artificial muscles, their potential, and their tradeoffs. For our concept as of today, we are planning to use translational drives. Translational drives directly couple the driving forces to the movement direction and they are commercially available. However, for all electric drives one challenge remains: the storage of energy. Fortunately (or unfortunately), this is also a big issue in the automotive industry for the construction of electric vehicles. Therefore, we think that it is likely to see great improvement in the storage technology in the near future. This would also facilitate the use of electric drives as active element in functional artificial muscles.

The passive damping element (PDE) develops forces during the contraction, which may exceed over a very short period of time, the muscle output forces several times over. The question remains whether there are comparable forces internal to the artificial muscle in other technical embodiments. Unfortunately, this is commonly not reported in the literature. In our approach, we use an electric drive to produce the damping forces that are in fact a nonpassive damper. Are there any materials or other approaches possible instead of using an electric drive as presented in this approach? A magnetorheological damping element, for example, [23]?

Fortunately, the serial element (SE) seems to be the simplest challenge for a technical representation. This element should imply nonlinear force-displacement characteristics. Even a steel rod would show the dynamic characteristics similar to that of the serial element predicted in theory. However, as a must-have, this element needs to incorporate damping characteristics, yet very small [17]. It is to see how the artificial muscle prototype behaves when including a serial element like that observed in biology and postulated in theory [11].

### 4.2. What is Gained Using This Approach?

Understanding biological muscle characteristics and design is of great interest in biological science. Muscle models in general help to mathematically formulate muscle characteristics. The structure of our model is in essence purely mechanical. Therefore, it can serve as a functional starting point of bionic muscle design. Phenomenological models based on biological experiments were the first to define muscle characteristics, for example, [16]. Constantly improving lab techniques allowed to observe muscle phenomena (even) in greater detail, for example, [5]. Microscopic muscle models deduced from basic assumptions of muscle structure and/or functional relationships of single variables come into play shortly after, for example, [24]. However, the benchmark of muscle dynamics used for those microscopic models is still the phenomenological Hill relation [16]. One approach just recently succeeded in defining the macroscopic muscle characteristics without the need of any phenomenological information. In contrast, this approach was validated against the well-known experiments instead of being based on it [11]. Here, we used those findings to build a technical muscle and succeeded in the reproduction of crucial characteristics of biological muscle. With this approach, now, the macroscopic model can be iteratively improved accompanied by the technical muscle. In that, technical models can partly replace biological experiments.

### 4.3. Hill-Type Models for Control

Hill-type muscle models, as an alternative to joint torque generators, have been implemented in (multibody) computer models in order to generate movement. In this regard different control theories, that is, physiologically motivated ones, for example, equilibrium point hypothesis [25, 26], virtual model control [27], and others described above, come into operation. Hence, multibody models with Hill-type muscles as actuators allow for using control theories to generate movement. This way, control approaches [28, 29] are quantitatively tested and relevant control parameters [30] are determined. Furthermore, existing and/or newly developed control theories are compared.

In this study, different control approaches, that is, no feedback, P controller, and PID controller (see Section 2), were implemented and compared in two different inverted pendulums models, that is, one with muscles and the other one with direct torque generators. From this comparison of control and actuators, it can be concluded that the implementation of muscle-like characteristics changes the model's inherent stability. Thus, it leads to a modification of successful control strategies to generate a similar movement. Furthermore, the presented arrangement of technical elements for the CE also allows for the investigation of structural changes in biological muscle used for movement control.

For further and more detailed conclusion, the presented approach will be implemented as muscle-like actuators in more complex (human) models to investigate (physiological motivated) control strategies and structural changes of muscle.

## Figures and Tables

**Table 1:** Muscle parameters (*A*, *B*, *F*_max⁡_, *l*_CE,opt_) determined in experiments (see reference) and muscle model parameters (*R*_PDE_, (1 − *κ*_*v*_)*D*_PDE_), respectively, calculated (see the appendix).

Muscle	*A* [N]	*B* [m/s]	*F*_max⁡_ [N]	*l*_ce,opt_ [m]	*R*_PDE_	(1 − *κ*_*v*_)*D*_PDE_	Reference
Piglet gastrocnemius	3.0	0.015	30.0	0.015	0.003	2200	[17]
Cat soleus	4.8	0.042	21.0	0.033	0.011	620	[18]
Cat tenuissimus	0.05	0.057	0.18	0.032	0.600	4	[19]
Rat gastrocnemius	2.68	0.042	13.4	0.013	0.167	386	[20]
Rat tibialis anterior	4.3	0.053	4.3	0.027	0.076	162	[19]
Frog sartorius	0.18	0.012	0.67	0.031	0.287	72	[19]

**Table 2:** Parameters for the muscle model were based on a human tibialis anterior muscle (see [21] for a detailed description). The muscle model used for the study was described in detail in [17].

*l*_CE,opt_	*F*_max⁡_	Δ*W*	*V*_CE_	*A*_*rel*⁡,0_	*B*_*rel*⁡,0_	*l*_SEE,0_	Δ*U*_SEE,nll_	Δ*U*_SEE,*l*_	Δ*F*_SEE,0_	*D*_SE_	*R*_SE_
0.1 m	10000 N	0.57	4.0	0.25	2.25 s^−1^	0.23 m	0.1825	0.073	10000 N	0.3	0.01

## Appendix

### A. Model Derivation

In order to end up with a hyperbolic relation, two further assumptions had to be made. First, the force of the PDE was assumed to be

(A.1) $$\begin{array}{c} F_{\text{PDE}}=d_{\text{PDE}}\cdot {\dot{l}}_{\text{PDE}}=d_{\text{PDE}}\cdot {\dot{l}}_{\text{AE}}=d_{\text{PDE}}\cdot \left({\dot{l}}_{\text{CE}}-{\dot{l}}_{\text{SE}}\right), \end{array}$$

where the damping coefficient of the PDE depends linearly on the current muscle force *F*_CE_ = *F*_SE_:

(A.2) $$\begin{array}{c} d_{\text{PDE}}\left(F_{\text{CE}}\right)=D_{\text{PDE},\max}\cdot \left(\left(1-R_{\text{PDE}}\right)\cdot \frac{F_{\text{CE}}}{F_{\text{AE},\max}}+R_{\text{PDE}}\right). \end{array}$$

*D*_PDE,max⁡ _ is the maximum (at *F*_CE_ = *F*_AE,max⁡_) and *R*_PDE_ the normalised (to *D*_PDE,max⁡_) minimum (force independent) value of *d*_PDE_(*F*_CE_). Second, the gearing ratio

(A.3) $$\begin{array}{c} \kappa_{v}=\frac{{\dot{l}}_{\text{SE}}}{{\dot{l}}_{\text{CE}}} \end{array}$$

between internal (SE) and external (muscle) velocities was represented by an arbitrary parameter value *κ*_*v*_.

The characteristics of the SE did not have to be specified. The AE is the source of mechanical energy. It may depend on length and on the macroscopic chemical state of the muscle, that is, the relative number of actively force-producing cross-bridges quantified by the normalised muscle activation 0 ≤ *q* ≤ 1.

In order to meet the conditions of our artificial muscle experiments presented in this paper, we had to modify the just reviewed model [11] with respect to only one feature. In contrast to  (6) in [11], which related the isometric force $F_{\text{CE}}({\dot{l}}_{\text{CE}}=0)=F_{\text{CE},0}$ (see (2) for ${\dot{l}}_{\text{CE}}=0$) as a linear function of contraction velocity ${\dot{l}}_{\text{CE}}$ to the AE force *F*_AE_, we now assume the identity

(A.4) $$\begin{array}{c} F_{\text{CE},0}=F_{\text{AE}}. \end{array}$$

Equation (A.4) is as consistent to the set of model equations (2), (3), (A.1), (A.2), and (A.3) as is (6) in [11] to this very set.

Substituting (A.1), the explicit dependency of *d*_PDE_(*F*_CE_) on force *F*_CE_ and model parameters (A.2), and (A.3) into (2) makes the latter force equilibrium (2) constitute a hyperbola

(A.5) $$\begin{array}{c} \left(F_{\text{CE}}+A\right)\cdot {\dot{l}}_{\text{CE}}=-B\cdot \left(F_{\text{CE},0}-F_{\text{CE}}\right) \end{array}$$

with the Hill parameters *A*, *B* and the isometric force *F*_CE,0_ being positive and ${\dot{l}}_{\text{CE}}$ consistently being negative in the shortening (concentric) case. The Hill parameters are

(A.6) A=RPDE1−RPDE·FAE,max⁡B=11−RPDE·11−κv·FAE,max⁡DPDE,max⁡=RPDE1−RPDE·FAE,max⁡FAE·l˙CE,max⁡,

with the corresponding maximum shortening velocity

(A.7) l˙CE,max⁡=BA·FCE,0=BArel⁡=1RPDE·11−κv·FAEDPDE,max⁡.

The unloaded contractile element (*F*_CE_ = 0) would contract concentrically with ${\dot{l}}_{\text{CE}}=-{\dot{l}}_{\text{CE},\max}$:

(A.8) $$\begin{array}{c} A_{\mathrm{rel}}=\frac{A}{F_{\text{CE},0}}=\frac{F_{\text{AE},\max}}{F_{\text{AE}}}\cdot \frac{R_{\text{PDE}}}{1-R_{\text{PDE}}}, \end{array}$$

which is defined as the Hill parameter *A* normalised to the current isometric force *F*_CE,0_ = *F*_AE_. Note that, for given *F*_CE,0_ = *F*_AE_, a concurrent parameter variation fulfilling *B*/*A* = const meets the constraint ${\dot{l}}_{\text{CE},\max}=\text{const}$, whereas solely the curvature is changed. In our model, this is equivalent to (1 − *κ*_*v*_) · *D*_PDE,max⁡_ · *R*_PDE_ = const.
